# Annotations

**Published:** 1889-12-28

**Authors:** 


### Annotations.

**Dabblers in Electricity.** It was never truer than at the present time that "fools rush in where angels fear to tread." Electricity is one of the most powerful and one of the most dangerous agents known to man. It is, in fact, not known to man. A few people here and there know a little about it, and that little makes them regard it with extreme respect. But the unknowing multitude are clamorous to try it in every conceivable way. They will use it for belts, for liver pads, for chest protectors, and nobody knows what, on the recommendation of anybody or nobody. The *British Medical Journal* calls attention to the fact that shocks to the nervous system, in persons of a nervous disposition, are not easily got over, but may induce what is known to doctors as "neurasthenia, or traumatic hysteria (so-called), which may remain for months or years." Many people suppose that the only kind of shock which can bring about serious and lasting consequences like these is that caused by some terrible accident, as of the fall of an overhead wire, or the like. But that is a very grave mistake. The writer knows at least one person who has been grievously and irreparably injured by the too persistent and energetic use of electricity medically applied. It was no fault of the patient, but of her physician; and he not a tyro, but a man of the highest standing as a specialist. He tried to effect quickly by electricity what could only be effected slowly by a real building up of the nervous and muscular energy of the body. The result was that the condition—loss of voice—which was temporary ten or twelve years ago, has been permanent ever since, with one or two brief exceptions, when it was removed by prolonged courses of steel and change of air. Not only has the loss of voice remained persistent, but the patient, a thoroughly sensible woman, suffers from the most painful depression of spirits, alternating with agonies of excessive nerve sensibility. She is for all practical purposes a "nervous wreck." Had she been of an excitable, instead of a placid disposition, it is possible that she would long ago have become uncontrollable, and, in short, a lunatic. It is evident, then, that electricity cannot be played with, either by physicians or patients. All men and women will be well advised not to have anything whatever to do with belts, or pads, or other electrical appliances, except on the recommendation of a medical man whose skill they can trust and whose honesty has been proved. Medical men may have some little natural prejudice against outsiders who practise any part of their art; but no worthy physician will seek to deprive his patient of any kind of treatment, by whomsoever advised, which he thinks likely to be of real service. There are plenty of trustworthy doctors, and it is much wiser to rely on their proved skill and honesty than on any outsider, however plausible and prosperous he may seem to be.

**Do Doctors Despise the Influenza Epidemic?** It is to be feared that English physicians look upon the present continental epidemic of influenza with a spice of mild contempt. Our medical contemporaries have made very little of it, and hardly a scientist of eminence has thought it worth while to express an opinion either as to its etiology or its treatment. A sort of languid interest has been excited among the microbe hunters; but even they do not seem to care whether the microbe of the disease, if there be one, has his natural habitat in the mucous membrane of the nose, and is now preternaturally active in the multiplication of himself, or whether he is ordinarily a denizen of the atmospheric air, and has been making raids for his pleasure into an excessive number of nostrils and throats. Our British doctors and professional newspapers are, as we have said, particularly indifferent. It accords with the national character. We hate exaggeration and sensationalism. There is no doubt that the foreign newspapers have made the most of this last new claim to distinction. The Parisian or Viennese who has not had an attack of influenza is probably guilty of as stupid a solecism as he who should go out to dine in his morning coat. Every man with the least nobility in his blood, or the faintest claim to social recognition, has undoubtedly had, or soon will have, the fashionable disease. No woman especially will think of missing it any more than she will go to the opera without her cloak, or to the Park without her riding-habit. In the meantime, the more lymphatic among our own fellow-countrymen are glad to be left "out in the cold." It may happen that the double infection of the actual disease and the craze for it will attack some of our apostles of fashionable form. In that case, our newspapers, too, will be full of "influenza" from day to day. Let us hope that some more rational and pleasant amusement will be found for British idlers of both sexes than "sneezing and sipping posset" this Christmas season. We do not put forward the contention that the influenza "epidemic" is what Carlyle would have called a "mere simulacrum," but so far as appears there is nothing which cannot be explained on the hypothesis of very unfavourable weather.

**Hereditary Sleeplessness.** "Now blessings light on him that first invented sleep!" wrote Cervantes; "it covers a man all over, thoughts and all, like a cloak; it is meat for the hungry, drink to the thirsty, heat for the cold, and cold for the hot." To which may be added that it is "the rarest medicine known to the pharmacopœias of the whole world." Without sleep the strongest-limbed giant loses his power, the mightiest intellect his wit, and the holiest saint his sanctity. The persistent and unremitting absence of sleep can only end in lunacy, in suicide, or in death. It is one of the most portentous facts in the life of this generation that sound sleep is becoming less and less common, both with men and women, and even with children. Physicians are often asked whether such and such a disease is hereditary? There is no doubt that most of the conditions which become permanent in one or both parents tend to reproduce themselves in children born after the establishment of the conditions in the parent. It is in this way that sleeplessness may, and often does, prove hereditary. But what a legacy to leave to son or daughter in a world where only the fittest are destined to survive! It is not, however, the question of survival that is the most grave in this relation. What a heritage of daily misery, often amounting to positive anguish, has he fallen heir to who loses his sleep on the smallest provocation! If the man is to be "blessed who first invented sleep," surely that age or that parent deserves the opposite of a benediction that inflicts on his descendants the curse of hereditary insomnia. From this point of view, fathers and mothers should be very resolute in seeking prompt restoration to health when they find that loss of sleep is one of their prominent symptoms. It is a condition that tends steadily to grow worse, and is only to be removed by the return to normal health. Happily for children, the hereditary tendency to sleeplessness, like so many other inherited tendencies, will wear itself out of the system under favourable conditions. It is one of the deepest obligations that parents owe their children to make sure that they are placed in the best possible circumstances for getting finally rid of so deadly a tendency as that to insomnia. The little ones should be brought up in the purest obtainable atmosphere. This is imperative. They should be fed with the wisest discrimination so that disorders of stomach, bowel, and liver may be entirely avoided; and their school work should be regulated with the utmost regard to their intellectual capacity and power of endurance. All brain work should cease for such children at the latest at six o'clock in the afternoon. If to these be added plenty of play and freedom from school or home worry, it may be hoped that normal sleep power will be attained with adult age. Let all readers understand that this is no alarmist doctor's "fad," but the setting forth of the sober facts of personal experience. Undoubtedly the increased development of brain and brain functions tends to the production of nervous disorders; and the only rational way of dealing with the new circumstances is to recognise them promptly, and to counteract them by intelligent regularity of living, and ordered moderation of work.

